# A Protocol is not Enough: Enhanced Recovery Program-Based Care and Clinician Adherence Associated with Shorter Stay After Colorectal Surgery

**DOI:** 10.1007/s00268-020-05810-w

**Published:** 2020-10-20

**Authors:** Ben E. Byrne, Omar D. Faiz, Alex Bottle, Paul Aylin, Charles A. Vincent

**Affiliations:** 1 Centre for Surgical Research, Population Health Sciences, Bristol Medical School, University of Bristol, Canynge Hall, 39 Whatley Road, Bristol, BS8 2PS UK; 2 Department of Surgery and Cancer, Imperial Patient Safety Translational Research Centre, Imperial College London, London, UK; 3 Surgical Epidemiology, Trials and Outcome Centre, St Marks Hospital, Harrow, UK; 4 Dr. Foster Unit, Department of Primary Care and Public Health, Imperial College London, London, UK; 5 Department of Experimental Psychology, University of Oxford, Oxford, UK

## Abstract

**Background:**

Randomised trials have shown an Enhanced Recovery Program (ERP) can shorten stay after colorectal surgery. Previous research has focused on patient compliance neglecting the role of care providers. National data on implementation and adherence to standardised care are lacking. We examined care organisation and delivery including the ERP, and correlated this with clinical outcomes.

**Methods:**

A cross-sectional questionnaire was administered to surgeons and nurses in August–October 2015. All English National Health Service Trusts providing elective colorectal surgery were invited. Responses frequencies and variation were examined. Exploratory factor analysis was performed to identify underlying features of care. Standardised factor scores were correlated with elective clinical outcomes of length of stay, mortality and readmission rates from 2013–15.

**Results:**

218/600 (36.3%) postal responses were received from 84/90 (93.3%) Trusts that agreed to participate. Combined with email responses, 301 surveys were analysed. 281/301 (93.4%) agreed or strongly agreed that they had a standardised, ERP-based care protocol. However, 182/301 (60.5%) indicated all consultants managed post-operative oral intake similarly. After factor analysis, higher hospital average ERP-based care standardisation and clinician adherence score were significantly correlated with reduced length of stay, as well as higher ratings of teamwork and support for complication management.

**Conclusions:**

Standardised, ERP-based care was near universal, but clinician adherence varied markedly. Units reporting higher levels of clinician adherence achieved the lowest length of stay. Having a protocol is not enough. Careful implementation and adherence by all of the team is vital to achieve the best results.

**Electronic supplementary material:**

The online version of this article (10.1007/s00268-020-05810-w) contains supplementary material, which is available to authorized users.

## Introduction

The Enhanced Recovery Program (ERP) is a complex, multidisciplinary peri-operative care package that can reduce length of stay after surgery [1–3]. Much research has examined adherence to ERP components and length of stay at the patient level [4–7]. While this approach is important, it overlooks the influence of the clinical team on outcomes and does not explain persistent outcome variation between units after risk adjustment. There is a lack of research in this area, and there are no national data on unit adoption of the ERP.

We developed a novel approach to explore implementation of the ERP, combined with broad examination of care organisation and delivery within colorectal units. This study distinguishes itself from previous ERP-focused research by gathering data at the unit—rather than patient level. Our pragmatic, higher-level approach asked participants about key ERP components. We situated this within a broad assessment of care within the unit, to explore a wide range of factors that may influence outcomes, based on previous work [8]. We aimed to measure care organisation and delivery, with particular focus on the ERP, and explore its association with clinical outcomes. Better understanding of variation in outcomes may guide future quality improvement interventions.

## Materials and methods

A cross-sectional survey of English National Health Service (NHS) Trusts providing colorectal surgery was conducted. Consultants and registered nurses were invited.

### Questionnaire design

The questionnaire comprised 8 sections (Table 1), based upon previous work [8]. Respondents rated agreement with statements about unit practice from 1 (strongly disagree) to 5 (strongly agree). Paper and online questionnaires were piloted with five research colleagues each.

**Table 1:** Questionnaire sections

Theme
Standardisation of care
Components of peri-operative care based upon the ERP
Organization of the clinical team for routine care
Monitoring of patients for post-operative deterioration
Clinical response to post-operative deterioration
Team functioning
Resources and staffing
Collection and use of clinical information

### Unit selection

All English colorectal units were approached through the National Institute for Health Research (NIHR) Coordinated System for gaining NHS Permissions. Subspecialist Trusts were excluded.

### Questionnaire administration

Colour-printed, personalised invites with a prepaid return envelope and 4-week reminder were sent to surgeons and senior ward nurses [9–11]. The survey was emailed to named colorectal specialist nurses who were encouraged to snowball to colleagues. Two reminders were emailed at 2-week intervals. The study closed 8 weeks after final invites. All responses were collected during August–October 2015.

### Outcomes

Unit-level length of stay, in-hospital mortality and 28-day readmission rates were obtained from Hospital Episode Statistics data by the Dr Foster Unit at Imperial College, supported through a research grant from Dr Foster Intelligence. Dr Foster’s routinely processed outcomes were risk-adjusted and standardised to the national average [12]. Hospital-level outcomes were retrieved for all elective colorectal resections between July 2013 and June 2015.

### Statistical analysis

Postal response rates were derived. Online response rates could not be determined due to the sampling strategy. Responses with over 10% missing items were excluded. Response frequencies were examined for questionnaire items to assess practice variation.

Exploratory factor analysis was performed using the SPSS R-menu v2.4 [13] with SPSS Statistics version 24 (IBM, Armonk, New York, USA). Questionnaire items with very low levels of variation were excluded due to the lack of discriminatory potential. Missing data were imputed with the median. Spearman's correlation was used [14]. The number of factors was determined using several techniques: the optimal coordinate (OC) approach, Horn’s parallel analysis (PA) technique, Velicer’s minimum average partial (MAP), the Very Simple Structure (VSS) criterion, and Ruscio and Roche’s Comparison Data (CD) [13]. Exploratory factor analysis was performed using principal axis factoring, oblique rotation (oblimin quartimin) and a factor loading cut-off of 0.4 [15, 16]. Suitability for analysis was tested with Bartlett’s test of sphericity and the Kaiser–Meyer–Olkin (KMO) statistic. The pattern matrix was inspected to interpret factors and examine cross-loadings. Items were considered for cohesion and meaning alongside other items for each factor and could be excluded if they lacked clinical coherence. Factor scores were calculated using weighted sum scores for items with loadings over 0.4 before standardisation to a maximum of 100. The distribution of factor scores and outcomes were examined using Q-Q plots, aiming to use parametric association tests if appropriate. Outliers were considered for exclusion.

For hospital analysis, units with less than 2 responses were excluded. Average hospital factor scores were examined for bivariate correlation with outcomes. Multiple regression assessed unique association between factors and outcomes. Given the novel exploratory nature of this work, results with *p-*value < 0.10 were examined and considered indicative of a possible relationship between variables.

As a national, observational study, no sample size calculation was appropriate.

## Results

### Response rates and completeness

**Fig. 1:**
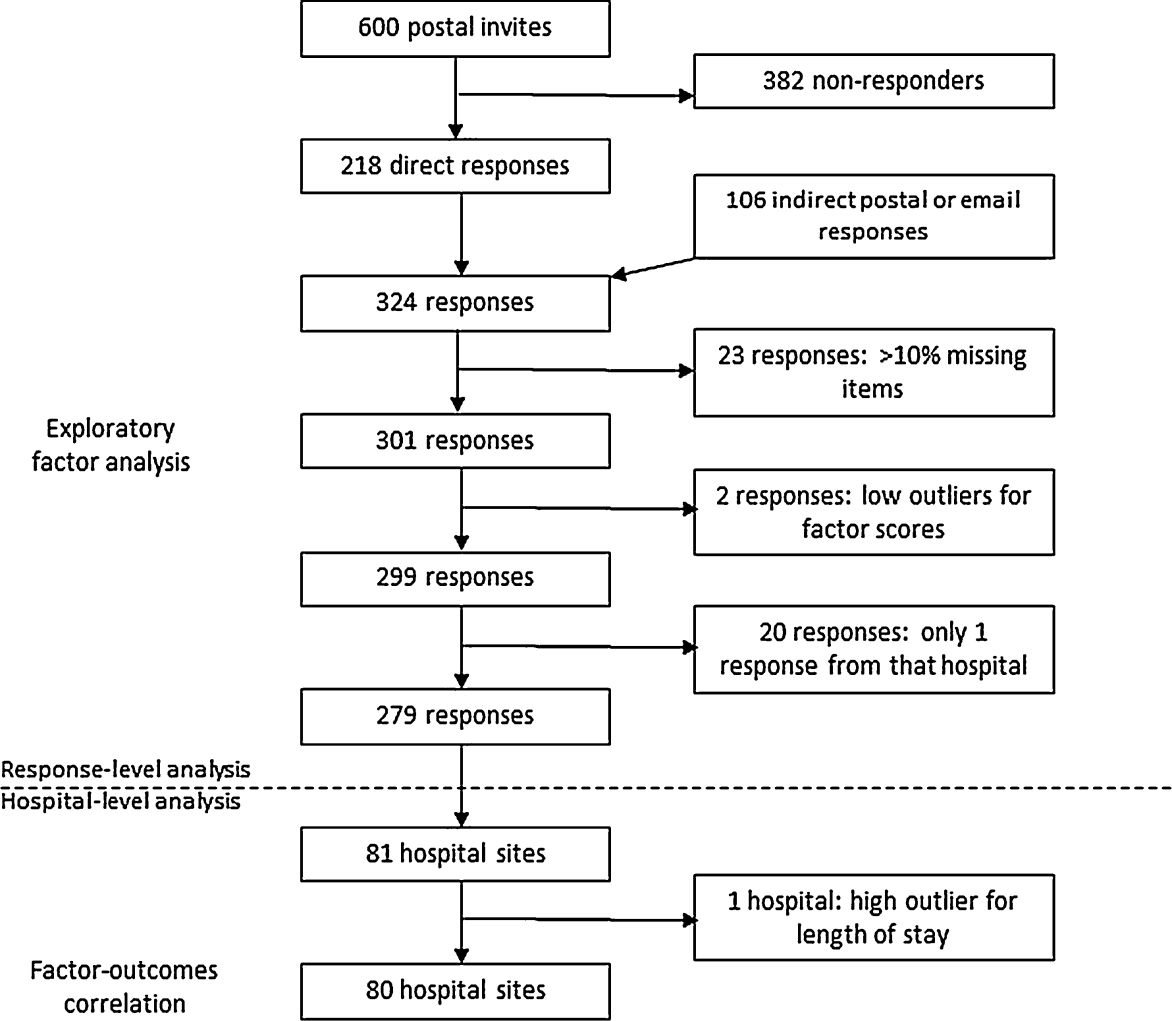
Flow chart of responses included in analyses

90/136 (66.2%) Trusts agreed to participate. At least, one survey was returned for 84/90 (93.3%) Trusts. 218/600 (36.3%) postal invites were returned by the intended recipient (Fig. 1). 6 mailed questionnaires were returned by specialist nurses. 100 online responses were received. After excluding responses with > 10% missing data, 301/324 (93.9%) datasets underwent factor analysis. 262/301 (87.0%) represented complete datasets. Among incomplete responses, 27 had 1 missing item and 12 had 2–4 missing items.

### Clinical practice

#### Standardisation and ERP-based care components

281/301 (93.4%) respondents agreed or strongly agreed that there was a defined management protocol for elective patients, such as an ERP, for their patients (see Supplementary Table 1 for full results). 278/301 (92.4%) participants agreed or strongly agreed that patients normally started drinking and/or eating within the first 24 h after surgery, and 277/300 (92.3%) indicated that they usually mobilised in the first 24 h. 190/300 (63.3%) of participants replied that elective patients rarely had abdominal drains or nasogastric tubes (NGTs).

Regarding departmental standardisation, 218/301 (72.4%) agreed or strongly agreed that all consultants followed the local protocol. Adherence by clinicians varied depending upon care component. 248/301 (82.4%) indicated all consultants managed post-operative mobilisation similarly, whereas only 181/300 (60.3%) agreed or strongly agreed that all consultants managed post-operative diet and fluids similarly.

#### Other aspects of care organisation and delivery

Participants were asked about routine care provision and complication detection. 175/300 (58.3%) participants agreed or strongly agreed most elective patients were seen daily by a consultant. 110/301 (36.5%) participants agreed or strongly agreed certain elements of routine care were nurse-led. 293/300 (97.7%) respondents agreed or strongly agreed they had an observation-based early warning score system to detect patient deterioration. When deterioration was suspected, 228/301 (75.7%) participants agreed or strongly agreed ward nurses were encouraged to escalate directly to the patient’s consultant if appropriate, regardless of the physiological parameters. Fewer respondents, 181/300 (60.3%), agreed or strongly agreed most ward nurses would feel comfortable calling the patient’s consultant directly.

Ratings of teamwork were broadly positive, with between 227/301 (75.4%) and 279/300 (93.0%) agreeing or strongly agreeing with statements about team functioning, including praise for hard work, having an open culture and good leadership. 123/298 (41.3%) of participants agreed or strongly agreed there was a good nurse-to-patient ratio for patient needs. 192/301 (63.8%) agreed or strongly agreed there was a good number of non-consultant doctors on the ward during weekday working hours, dropping to 108/298 (36.2%) for out-of-hours.

### Factor analysis

Due to very high levels of agreement or strong agreement, 2 survey items were excluded from factor analysis: having a clearly defined written management protocol and having an observation-based early warning score. 4 factors were indicated by OC, PA, Velicer’s squared MAP and VSS. Velicer’s fourth power MAP suggested 5 factors, and CD suggested one. Therefore, factor analysis was performed with 4 factors. The rotated pattern matrix is shown in Table 2. There were no item cross-loadings. The KMO statistic was good (KMO = 0.880), and Bartlett’s test of sphericity was strongly significant (*p* < 0.001), indicating suitability for factor analysis. Factor 1 represented teamwork and communication between consultants and nurses. Factor 2 represented ERP-based care standardisation and clinician adherence. There was no separation of items examining specific aspects of clinical care (e.g. use of abdominal drains or nasogastric tubes), and items about uniform adherence by all consultants. Factor 3 represented ward staffing levels, and factor 4 represented support for complication management. Q-Q plots revealed 2 very low outlying survey responses: one for factor 1; one for both factors 2 and 4. These were excluded from further analysis.

**Table 2:** Exploratory factor analysis rotated pattern matrix with item loadings for each factor

	F1	F2	F3	F4
In the colorectal unit, there is good leadership with a balance between long-term plans and short-term targets and goals	0.804			
In the colorectal unit, hard work, good practice and good performance are praised and supported	0.772			
In the colorectal unit, there is an open culture and willingness to discuss and learn from errors	0.722			
The quality of teamwork and communication between the colorectal consultants and nurses is very good	0.554			
There is regular feedback of information on how the colorectal team is performing to ward staff (e.g. regular information on length of stay and complication rates)	0.461			
Most ward nurses would feel comfortable calling a patient’s consultant directly if they felt it appropriate	0.459			
In the colorectal unit, ward nurses are trained and encouraged to recognise deterioration and complications in patients, outside the use of observations and early warning scores (e.g. using changes in symptoms such as abdominal pain or vomiting)	0.429			
Ward nurses are encouraged to escalate directly to a patient’s consultant if they judge it appropriate, regardless of the observations or early warning score	0.417			
Elective patients normally* begin drinking and/or eating* within the first 24 h after surgery		0.739		
The consultants providing elective surgery all manage post-operative* oral intake of fluids and diet* the same way		0.688		
All the consultants providing elective colorectal surgery follow a clear protocol to guide day-to-day management		0.671		
The team normally follows pre-defined criteria when discharging elective patients		0.628		
The consultants providing elective surgery all manage post-operative* mobilisation* the same way		0.612		
Elective patients normally* mobilise* within the first 24 h after surgery		0.601		
Patients undergoing open surgery receive similar care to patients undergoing laparoscopic surgery (e.g. oral intake and mobilisation)		0.592		
All elective patients receive standardised preoperative counselling		0.535		
All elective patients receive* detailed* preoperative counselling (e.g. pain management, mobilisation, eating and drinking, likely time to discharge)		0.530		
Elective patients very rarely have* abdominal drains or nasogastric tubes* after surgery		0.501		
After discharge, patients are followed up within the first 2 weeks (e.g. by phone or in clinic)		0.420		
For colorectal patients, there is a good number of non-consultant medical staff* during routine working hours* (i.e. Foundation Doctors to Registrars; Monday to Friday, 08.00–17.00)			0.618	
For colorectal patients, there is a good number of non-consultant medical staff* during out-of-hours* (i.e. Foundation Doctors to Registrars; overnight Monday to Friday and weekends)			0.519	
On the colorectal ward, there is a good nurse-to-patient ratio considering the needs of the patients			0.412	
If a post-operative patient deteriorates and needs a CT or ultrasound scan, this is normally done within 24 h				0.741
If a post-operative patient deteriorates and needs intensive care, the intensive care team can normally find a bed and transfer the patient promptly (e.g. severe chest infection with sepsis)				0.449
If a post-operative patient develops a leak from a bowel anastomosis and urgently needs to go back to theatre, they normally get their operation within 6 h				0.420
If a post-operative patient needs a drain inserting for an abdominal collection or abscess detected on a scan, this is normally done within 24 h of diagnosis (usually in interventional radiology)				0.402

### Hospital analysis

53 456 colorectal resections were included, with 489 (0.9%) in-hospital deaths, and 7 129 (13.3%) 28-day readmissions. Q-Q plots revealed one very high outlier for length of stay which was excluded from further analysis.

Factor scores were averaged across hospitals. 20 hospitals with only 1 survey response were excluded, leaving 279 responses from 81 sites. As Q-Q plots indicated suitability for parametric testing, factors and outcomes were examined for association using Pearson correlation (Table 3). Higher ratings of teamwork and communication in a hospital were significantly correlated with ERP standardisation and clinician adherence (*r* = 0.473, *p* < 0.001) and greater support for complication management (*r* = 0.368, *p* = 0.001). In addition, higher levels of ERP standardisation and clinician adherence were separately associated with greater support for complication management (*r* = 0.361, *p* = 0.001).

**Table 3:** Pearson correlation between hospital average standardised factor scores and risk-adjusted outcomes

	F1–teamwork and communication		F2–ERP standardisation and clinician adherence		F3–ward staffing		F4–complication management support		Length of stay		Mortality	
	R	*p*	r	*p*	r	*p*	r	*p*	r	*p*	r	*p*
F1–teamwork and communication	–	–	**.473**	**< .001**	.187	.096	**.368**	**.001**	−.159	.160	−.101	.375
F2–ERP standardisation and clinician adherence	**.473**	**< .001**	–	–	.147	.193	**.361**	**.001**	**−.301**	**.007**	−.219	.051
F3–ward staffing	.187	.096	.147	.193	–		.162	.152	.005	.962	−.133	.239
F4–complication management support	**.368**	**.001**	**.361**	**.001**	.162	.152	–	–	.024	.829	−.057	.614
Length of stay	−.159	.160	**−.301**	**.007**	.005	.962	.024	.829	–		**.337**	**.002**
Mortality	−.101	.375	−.219	.051	−.133	.239	−.057	.614	**.337**	**.002**	–	
Readmissions	.130	.249	−.024	.832	**−.254**	**.023**	−.130	.249	−.092	.416	.162	.151

*p* value–two-tailed significance test

Bold values indicate statistical significance (*p* < 0.05)

Higher ratings of ERP standardisation and clinician adherence were significantly associated with reduced length of stay (*r* = -–0.301, *p* = 0.007). There was weak, borderline significant association between higher ERP standardisation and clinician adherence and lower mortality rates (*r* = -–0.219, *p* = 0.051). Higher ratings of ward staffing were weakly significantly associated with lower rates of readmission at 28 days (*r* = -0.254, *p* = 0.023).

On multiple regression, the relationship between higher ratings of ERP standardisation and clinician adherence and shorter length of stay persisted (standardised beta = -0.334, *p* = 0.010; Table 4). The association between higher ward staffing levels and lower readmission rates was also reproduced. A new weak association emerged between higher levels of teamwork and communication and increased readmission rates (*r* = 0.266, *p* = 0.040).

**Table 4:** Multiple linear regression results examining hospital average standardised factors cores and risk-adjusted outcomes

	Length of stay	*p*	Mortality	*p*	Readmission	*p*
F1 – teamwork and communication	−.069	.593	.011	.933	**.266**	**.040**
F2 – ERP standardisation and clinician adherence	**−.334**	**.010**	−.221	.095	-.050	.692
F3 – ward staffing	.041	.715	−.108	.347	**−.270**	**.017**
F4 – complication management support	.164	.178	.036	.772	−.166	.169

Standardised beta coefficients provided

## Discussion

This is the first study to examine adoption of standardised, ERP-based care for colorectal surgery at the national level. Nearly all units reported having implemented this approach. Despite this, respondents reported wide variation in certain aspects of ERP-related care. Only 63.5% indicated that the team rarely used abdominal drains or nasogastric tubes. In addition, there was wide variation in reported clinician adherence to the ERP. Clinician adherence was lowest for oral intake, with only 60.5% of consultants managing post-operative oral intake similarly. Greater hospital ERP-based standardisation and *clinician* adherence were significantly associated with shorter length of stay. This key finding extends the evidence on the effects of the ERP beyond the confines of randomised controlled trials to national implementation across a healthcare system. Our findings also provide insights into how the ERP may have its effect to achieve the best possible outcomes.

The authors are aware of only one other study examining large-scale adoption of the ERP care. A survey of members of the Society of American Gastroenterological and Endoscopic Surgeons (SAGES) was limited by its focus on society members and very low response rate (4.5%, 229/5133), and did not correlate responses with outcomes [18]. The majority (70.4%) did not have an institutional ERP, suggesting care was not standardised within departments. By contrast, our findings document near-universal adoption of protocolised, ERP-based care in the English NHS.

This study complements the firm evidence-base supporting the ERP in reducing length of stay, based on systematic reviews and meta-analyses of randomised trials [1–3]. Our study examined real-world practice across a national healthcare system. Outside the rigors and resources of RCTs, we found robust association between higher reported levels of standardisation and adherence to the ERP, and shorter length of stay. External validity is often neglected and cannot be assumed [19]. We have demonstrated the successful generalisation and translation of a complex intervention, based on sound evidence, into widespread practice.

This study yields insights into ways the ERP may work. On factor analysis, individual ERP elements, such as early oral intake and avoiding abdominal drains, could not be separated from clinician adherence to ERP components. The shortest length of stay was achieved in hospitals where all consultants adhered to the protocol, as well as adopting the individual clinical components of the ERP. Almost all units reported having adopted protocolised care. The phenomenon of clinician adherence goes beyond having a written protocol. This builds upon our previous study which found that shortest stay was achieved if consultants or well-supported nurses were driving forward patient care [8]. Recognition of this human element is vital in understanding how interventions have their effects, and may be particularly important with complex, diffuse, multi-component interventions that work at different levels, such as the ERP [20].

Other findings suggest other benefits, and possible mechanistic mediators, of implementing a standardised protocol. Previous research on surgical teams has focused heavily on the operating theatre. A large-scale study in American Veterans Affairs hospitals showed team training reduced post-operative mortality rates [21]. However, other studies have had less encouraging results [22, 23]. When an ERP is introduced, clinical teams meet to discuss protocol details and spend time promoting awareness across the multi-professional team. We found hospitals with higher reported levels of ERP adoption and clinician adherence also reported higher levels of teamwork and consultant-nurse communication. Improved teamwork and communication may be a benefit of ERP implementation, or an indirect mediator of its effect, but the lack of independent association with clinical outcomes suggests it may not have been a direct factor in improving results in the current analysis.

Research on failure-to-rescue has highlighted the importance of complication management in surgical patients [24–26]. Higher reported levels of ERP standardisation and clinician adherence were also associated with greater reported support for complication management, such as prompt access to percutaneous drainage of an intra-abdominal abscess. However, as with teamwork and communication, there was no independent correlation with outcomes. Perhaps units that have worked together across professional groups to implement a successful ERP, tend to have higher levels of teamwork and communication, and are more effective in working with radiologists, intensive care and theatres to manage complications promptly. However, there was no association between these 3 factors and ward staffing levels. This may suggest that quality of teamwork is more important than having more members.

The trend towards lower mortality rates associated with higher reported levels of ERP standardisation and clinician adherence remained of borderline significance on regression analysis. Evidence on the impact of the ERP on mortality is mixed. Large, non-randomised series have reported an association between greater ERP adherence and reduced long-term mortality rates [27, 28]. However, data from meta-analyses of randomised trials report that the ERP is not significantly associated with reduced mortality rates [1–3]. The other associated features of units with higher levels of ERP standardisation and adherence, with a trend to better teamwork and support for complication management, provide a plausible mechanism by which lower mortality rates may be achieved. However, further in-depth work exploring variation in mortality rates is needed.

This study has important strengths and limitations. Over half of all Trusts approached took part. The questionnaire was built on previous qualitative research and underwent external review. Responses were collected from surgeons and nurses, mitigating biases of professional groups. We examined selected care elements and higher-level care organisation, avoiding a reductionist approach, and minimising the burden on responders. Questionnaire data are limited by well-known biases, including non-response bias [29]. It was not possible to compare responders and non-responders. Participants may have exhibited other biases, such as social desirability bias, answering questions in ways considered more socially acceptable, providing favourable assessments of care. Questionnaire responses reflect participants’ evaluation of practice and may differ from direct observations of clinical care. The study used a novel questionnaire which has not been evaluated for validity and reliability. However, the reported associations between responses and outcomes strongly support that questionnaire responses provided a valid measure of practice. While the data are now some years old, the findings of variation in practice, and association between ERP-based care, clinician adherence to protocolised care and clinical outcomes at unit level are still likely to be relevant, even if the exact details of care have changed over time. Organisational and cultural factors underlying the current findings may be specific to practice within the English NHS. However, the key finding of this study that standardised, ERP-based care and clinician adherence was associated with shorter stay, is likely to be relevant in similar Western countries.

This study has adopted a novel approach to understanding variation in surgical outcomes. Using a national, cross-sectional questionnaire and routine administrative data, we have demonstrated that higher reported levels of ERP-based care standardisation and clinician adherence were associated with shorter stay across a large sample of hospitals. By examining clinician adherence, we have highlighted the crucial agency of the clinical team in delivering excellent outcomes. We have shown that the ERP has been effectively implemented at scale in the English NHS. However, having a protocol is not enough. Careful implementation and adherence by all of the team is vital to achieve the best results.

## Electronic supplementary material

Below is the link to the electronic supplementary material.

Supplementary file1 (DOCX 32 kb)

## Abbreviations

CT: Computed tomography
ERP: Enhanced Recovery Program
NHS: National Health Service
NIHR: National Institute for Health Research
NGT: Nasogastric tube
USS: Ultrasound scan
