# Supplementary material for: A Protocol is not Enough: Enhanced Recovery Program-Based Care and Clinician Adherence Associated with Shorter Stay After Colorectal Surgery
Source: World J Surg. 2020 Oct 20;45(2):347–55. doi: 10.1007/s00268-020-05810-w (PMC7773614; doi:10.1007/s00268-020-05810-w)
Supplement: Supplementary file 1 — Supplementary file1 (DOCX 32 kb) [file 268_2020_5810_MOESM1_ESM.docx]

Supplementary Table 1. Frequency of responses to individual survey items.

|  | Strongly disagree | Disagree | Neither agree nor disagree | Agree | Strongly agree | n |
| --- | --- | --- | --- | --- | --- | --- |
| **Standardization of care** |  |  |  |  |  |  |
| There is a clearly defined, written management protocol for elective patients. (E.g. an Enhanced Recovery Program.) | 1 | 9 | 10 | 78 | 203 | 301 |
| All elective patients receive standardized preoperative counselling. | 1 | 10 | 17 | 120 | 153 | 301 |
| All the consultants providing elective colorectal surgery follow a clear protocol to guide day-to-day management. | 3 | 36 | 44 | 138 | 80 | 301 |
| The consultants providing elective surgery all manage postoperative oral intake of fluids and diet the same way. | 7 | 71 | 41 | 116 | 65 | 300 |
| The consultants providing elective surgery all manage postoperative mobilization the same way. | 3 | 26 | 24 | 129 | 119 | 301 |
| **Components of perioperative care** |  |  |  |  |  |  |
| All elective patients receive detailed preoperative counselling. (E.g. pain management, mobilization, eating and drinking, likely time to discharge.) | 1 | 13 | 20 | 137 | 129 | 300 |
| At the Multi-Disciplinary Team meeting for colorectal cancer patients, there is good attendance and input from all disciplines. | 2 | 10 | 19 | 75 | 190 | 296 |
| Elective patients very rarely have abdominal drains or nasogastric tubes after surgery. | 3 | 45 | 62 | 98 | 92 | 300 |
| Elective patients normally begin drinking and/or eating within the first 24 hours after surgery. | 1 | 7 | 15 | 129 | 149 | 301 |
| Elective patients normally mobilize within the first 24 hours after surgery. | 1 | 7 | 15 | 129 | 148 | 300 |
| Patients undergoing open surgery receive similar care to patients undergoing laparoscopic surgery. (E.g. oral intake and mobilization.) | 1 | 26 | 25 | 135 | 114 | 301 |
| The team normally follows pre-defined criteria when discharging elective patients. | 2 | 29 | 61 | 117 | 92 | 301 |
| After discharge, patients are followed up within the first 2 weeks. (E.g. by phone or in clinic.) | 8 | 41 | 30 | 93 | 128 | 300 |
| **Organization of team for routine care** |  |  |  |  |  |  |
| Elective colorectal patients are managed on a ward that mainly takes colorectal patients. | 16 | 52 | 22 | 94 | 117 | 301 |
| Elective colorectal patients are managed on a ward that mainly takes elective surgical patients. | 13 | 67 | 38 | 96 | 86 | 300 |
| It is clear which member of the team will make routine management decisions each day. | 3 | 19 | 42 | 129 | 107 | 300 |
| Normally there are delays in routine management while decisions are checked with consultants. | 50 | 156 | 55 | 33 | 6 | 300 |
| From Monday to Friday, the majority of elective patients are seen daily by a consultant. | 20 | 58 | 47 | 99 | 76 | 300 |
| Normally from Monday to Friday, Foundation Doctors or Core Trainees (House Officers or Senior House Officers) are the most senior person leading the daily ward round of elective patients. | 166 | 90 | 17 | 15 | 11 | 299 |
| Nurses are allowed to make well-defined changes in routine management without medical input. (I.e. they are nurse-led.) | 23 | 91 | 77 | 91 | 19 | 301 |
| **Monitoring patients for postoperative deterioration** |  |  |  |  |  |  |
| We have an observation-based early warning score system. Observation parameters are given a number and added together to create a score, to detect when patients are deteriorating. | 1 | 0 | 6 | 54 | 239 | 300 |
| Generally in the colorectal unit, nurses rely heavily on observations (and early warning scores if used) to determine if a patient is deteriorating. | 4 | 37 | 67 | 134 | 59 | 301 |
| In the colorectal unit, ward nurses are trained and encouraged to recognize deterioration and complications in patients, outside the use of observations and early warning scores. (E.g. using changes in symptoms such as abdominal pain or vomiting.) | 4 | 11 | 43 | 152 | 90 | 300 |
| **Response to postoperative deterioration** |  |  |  |  |  |  |
| Ward nurses follow a clear escalation protocol to obtain review of a deteriorating patient, based on observations or early warning scores. | 0 | 6 | 18 | 135 | 142 | 301 |
| Ward nurses are encouraged to escalate directly to a patient's consultant if they judge it appropriate, regardless of the observations or early warning score. | 4 | 30 | 39 | 116 | 112 | 301 |
| Most ward nurses would feel comfortable calling a patient's consultant directly if they felt it appropriate. | 9 | 38 | 72 | 112 | 69 | 300 |
| If a postoperative patient deteriorates and needs a CT or ultrasound scan, this is normally done within 24 hours. | 1 | 2 | 11 | 112 | 175 | 301 |
| If a postoperative patient needs a drain inserting for an abdominal collection or abscess detected on a scan, this is normally done within 24 hours of diagnosis. (Usually in Interventional Radiology.) | 3 | 20 | 51 | 142 | 83 | 299 |
| If a postoperative patient deteriorates and needs Intensive Care, the Intensive Care team can normally find a bed and transfer the patient promptly. (E.g. severe chest infection with sepsis.) | 3 | 11 | 29 | 138 | 119 | 300 |
| If a postoperative patient develops a leak from a bowel anastomosis and urgently needs to go back to theatre, they normally get their operation within 6 hours. | 2 | 10 | 38 | 142 | 108 | 300 |
| **Team functioning** |  |  |  |  |  |  |
| The quality of teamwork and communication between the colorectal consultants and nurses is very good. | 1 | 3 | 17 | 164 | 115 | 300 |
| In the colorectal unit, hard work, good practice and good performance are praised and supported. | 4 | 19 | 48 | 144 | 86 | 301 |
| In the colorectal unit, there is an open culture and willingness to discuss and learn from errors. | 1 | 10 | 31 | 146 | 111 | 299 |
| In the colorectal unit, there is good leadership with a balance between long-term plans and short-term targets and goals. | 3 | 16 | 55 | 154 | 73 | 301 |
| **Resources and staffing** |  |  |  |  |  |  |
| On the colorectal ward, there is a good level and quality of equipment (e.g. IV pumps, BP machines), as well as good provision of consumables (e.g. fluid bags, catheters). | 1 | 7 | 22 | 178 | 93 | 301 |
| In theatres, there is a good level and quality of technical equipment (e.g. modern laparoscopic stacks, surgical instruments), as well as good provision of consumables (e.g. staplers, sutures). | 1 | 12 | 54 | 131 | 83 | 281 |
| On the colorectal ward, there is a good nurse-to-patient ratio considering the needs of the patients. | 11 | 69 | 95 | 103 | 20 | 298 |
| For colorectal patients, there is a good number of non-consultant medical staff during routine working hours. (I.e. Foundation Doctors to Registrars; Monday to Friday, 08.00-17.00). | 8 | 30 | 71 | 147 | 45 | 301 |
| For colorectal patients, there is a good number of non-consultant medical staff during out-of-hours. (I.e. Foundation Doctors to Registrars; overnight Monday to Friday and weekends). | 16 | 67 | 107 | 90 | 18 | 298 |
| **Information collection and use** |  |  |  |  |  |  |
| There is reliable collection of performance data for colorectal patient care. (E.g. systematic submission of data for all colorectal cancer patients to the National Bowel Cancer Audit Program). | 10 | 10 | 43 | 114 | 120 | 297 |
| There is regular feedback of information on how the colorectal team is performing to ward staff. (E.g. regular information on length of stay and complication rates.) | 22 | 75 | 80 | 74 | 46 | 297 |
